# Using the Standing and Walking Assessment Tool at Discharge Predicts Community Outdoor Walking Capacity in Persons With Traumatic Spinal Cord Injury

**DOI:** 10.1093/ptj/pzad106

**Published:** 2023-08-10

**Authors:** Heather A Hong, Kristen Walden, James J Laskin, Di Wang, Dilnur Kurban, Christiana L Cheng, Lindsey Guilbault, Erica Dagley, Chelsea Wong, Shane McCullum, Dany H Gagnon, Jean-François Lemay, Vanessa K Noonan, Kristin E Musselman, The Canadian SCI Standing and Walking Measures Group

**Affiliations:** Praxis Spinal Cord Institute, Vancouver, British Columbia, Canada; Praxis Spinal Cord Institute, Vancouver, British Columbia, Canada; Praxis Spinal Cord Institute, Vancouver, British Columbia, Canada; Praxis Spinal Cord Institute, Vancouver, British Columbia, Canada; Praxis Spinal Cord Institute, Vancouver, British Columbia, Canada; Praxis Spinal Cord Institute, Vancouver, British Columbia, Canada; Parkwood Institute, London, Ontario, Canada; Nova Scotia Rehabilitation and Arthritis Centre, Halifax, Nova Scotia, Canada; Toronto Rehabilitation Institute, University Health Network, Toronto, Ontario, Canada; Stan Cassidy Centre for Rehabilitation, Fredericton, New Brunswick, Canada; School of Rehabilitation, Faculty of Medicine, Université de Montréal, Montréal, Québec, Canada; CIUSSS du Centre-Sud-de-l'Île-de-Montréal, Site Institut Universitaire sur la Réadaptation en Déficience Physique de Montréal, Montréal, Québec, Canada; School of Rehabilitation, Faculty of Medicine, Université de Montréal, Montréal, Québec, Canada; CIUSSS du Centre-Sud-de-l'Île-de-Montréal, Site Institut Universitaire sur la Réadaptation en Déficience Physique de Montréal, Montréal, Québec, Canada; Praxis Spinal Cord Institute, Vancouver, British Columbia, Canada; Department of Physical Therapy and Rehabilitation Sciences Institute, Temerty Faculty of Medicine, University of Toronto, Ontario, Canada; KITE Research Institute, Toronto Rehabilitation Institute, University Health Network, Toronto, Ontario, Canada

**Keywords:** Ambulation, Functional Outcome, Prediction, Rehabilitation, SCIM III, Spinal Cord Injury, Standing and Walking Assessment Tool

## Abstract

**Objective:**

The Standing and Walking Assessment Tool (SWAT) standardizes the timing and content of walking assessments during inpatient rehabilitation by combining 12 stages ranging from lowest to highest function (0, 0.5, 1A, 1B, 1C, 2A, 2B, 2C, 3A, 3B, 3C, and 4) with 5 standard measures: the Berg Balance Scale, the modified Timed “Up & Go” test, the Activities-specific Balance Confidence Scale, the modified 6-Minute Walk Test, and the 10-Meter Walk Test (10MWT). This study aimed to determine if the SWAT at rehabilitation discharge could predict outdoor walking capacity 1-year after discharge in people with traumatic spinal cord injury.

**Methods:**

This retrospective study used data obtained from the Rick Hansen Spinal Cord Injury Registry from 2014 to 2020. Community outdoor walking capacity was measured using the Spinal Cord Independence Measure III (SCIM III) outdoor mobility score obtained 12 (±4) months after discharge. Of 206 study participants, 90 were community nonwalkers (ie, SCIM III score 0–3), 41 were community walkers with aids (ie, SCIM III score 4–6), and 75 were independent community walkers (ie, SCIM III score 7–8). Bivariate, multivariable regression, and an area under the receiver operating characteristic curve analyses were performed.

**Results:**

At rehabilitation discharge, 3 significant SWAT associations were confirmed: 0–3A with community nonwalkers, 3B/higher with community walkers with and without an aid, and 4 with independent community walkers. Moreover, at discharge, a higher (Berg Balance Scale, Activities-specific Balance Confidence Scale), faster (modified Timed “Up & Go,” 10MWT), or further (10MWT) SWAT measure was significantly associated with independent community walking. Multivariable analysis indicated that all SWAT measures, except the 10MWT were significant predictors of independent community walking. Furthermore, the Activities-Specific Balance Confidence Scale had the highest area under the receiver operating characteristic score (0.91), demonstrating an excellent ability to distinguish community walkers with aids from independent community walkers.

**Conclusion:**

The SWAT stage and measures at discharge can predict community outdoor walking capacity in persons with traumatic spinal cord injury. Notably, a patient’s confidence in performing activities plays an important part in achieving walking ability in the community.

**Impact:**

The discharge SWAT is useful to optimize discharge planning.

## Introduction

Validated outcome measures allow rehabilitation professionals to evaluate an individual’s functional ability, plan treatment, and set appropriate rehabilitation goals.^1^^,^^2^ They enable clinicians to objectively monitor progress and assess rehabilitation impact, thereby supporting clinical decision-making, quality assurance, and clinical research.^3^^,^^4^ Several measures are available for standing/walking following a spinal cord injury (SCI), each with distinct strengths and limitations^5^^,^^6^; however, there is little consensus between rehabilitation professionals on which to use and when to use them. For example, 1 study reported that >50% of responding physical therapists of the American Physical Therapy Association did not regularly use standardized measures,^4^ and another study from Saudi Arabia reported that 39% of rehabilitation professional participants did not use standardized measures.^3^

**Figure 1:**
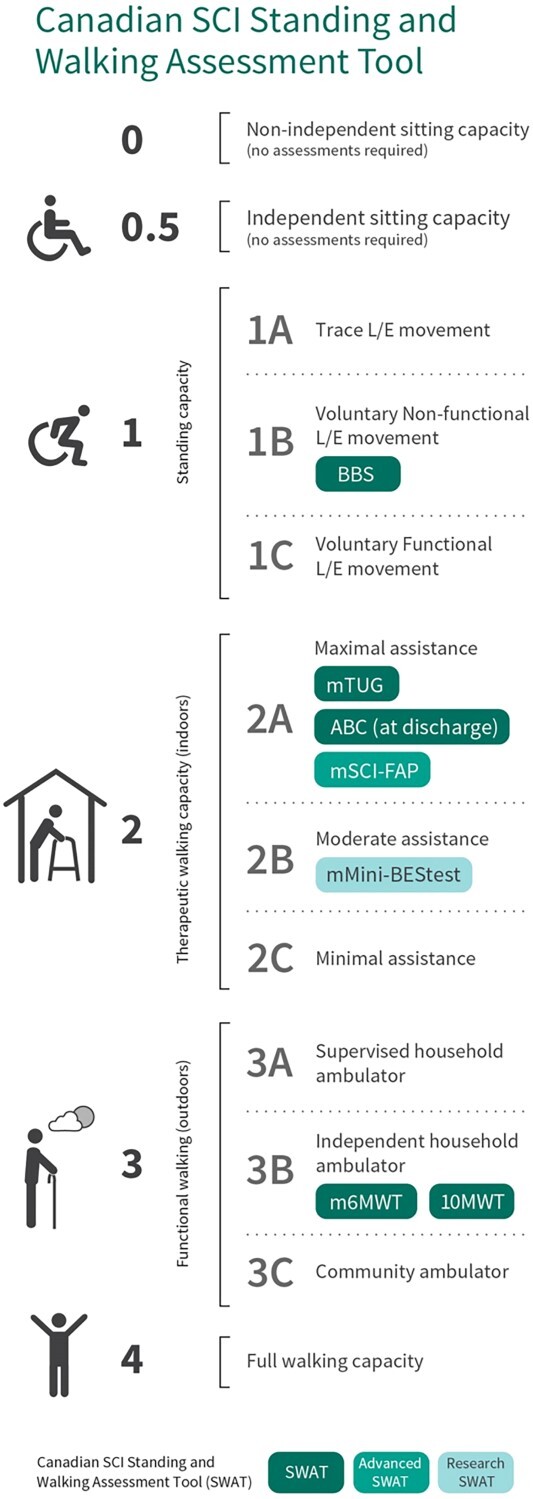
The Standing and Walking Assessment Tool (SWAT). Adapted with permission from Verrier M, Gagnon D, Musselman K. Toolkit for SCI Standing and Walking Assessment. Version 3.0. Rick Hansen Institute/Rick Hansen Spinal Cord Injury Registry (RHSCIR); February 2016.^1^ There are 3 levels of SWAT collection for a rehabilitation facility to choose from, depending on feasibility and goals in their local context ie, Basic, Advanced, or Research SWAT. The basic SWAT measures include the Berg Balance Scale (BBS), modified Timed “Up & Go” (mTUG), Activities-Specific Balance Confidence (ABC) Scale, modified 6-Minute Walk Test (m6MWT), and the 10-Meter Walk Test (10MWT). Additional measures include the modified Spinal Cord Injury Functional Ambulation Profile (mSCI-FAP) and the mini-Balance Evaluation Systems Test (mini-BESTest) which are performed in the Advanced and Research SWAT, respectively.

Variability in use, timing, and choice of measures can limit comparison across individuals, facilities, and the ability to pool data to inform health care policies. Thus, in 2012, the Standing and Walking Assessment Tool (SWAT) was developed by the Canadian SCI Standing and Walking Measures Group (physical therapists and clinical researchers) to support the standardization of standing and walking assessments during inpatient rehabilitation.^1^^,^^7^ SWAT is a tool that combines stages of standing and walking ability with preexisting measures for walking and balance.^1^ There are 3 levels of SWAT collection for a rehabilitation facility to choose from ie, Basic, Advanced, or Research (Fig. 1). Depending on their local context, rehabilitation facilities will collect their chosen level of SWAT on all of their clients with SCI. For this study, we focused on SWAT Basic.

The 12 SWAT stages are based on the individual’s functional ability ranging from 0 (non-independent sitting capacity), to 4 (full walking capacity) (Fig. 1). Stages 1 to 3 are further subdivided into A, B, and C based on the level of lower extremity movement, level of assistance required, and by the level of walking distance achievable, respectively (Suppl. Tab. 1). The SWAT stages were shown to be valid^7^^,^^8^ and responsive^8^ in the subacute traumatic SCI (TSCI) population. The SWAT measures include the Berg Balance Scale (BBS),^9^ modified Timed “Up & Go” (mTUG),^10^ Activities-Specific Balance Confidence (ABC) Scale,^11^ modified 6-Minute Walk Test (m6MWT),^10^ and 10-Meter Walk Test (10MWT).^12^

At rehabilitation admission, an individual is assessed to determine which SWAT stage he/she is at, and the assigned measure/s at and above that threshold stage are performed. Upon discharge, the individual’s functional ability is reassessed, and the measure/s at and above that SWAT threshold stage are repeated (Fig. 1). For example, if an individual is admitted, and they have the ability to stand independently with minimal assistance from a gait aid but are unable to voluntarily generate reciprocal steps, the individual is classified SWAT stage 1C and will have the BBS completed. Upon discharge, if that individual is able to walk with moderate physical assistance, they would be classified as SWAT stage 2B, and the BBS, mTUG, and ABC scale would be completed. Thus, the SWAT stages facilitate the clinicians’ selection of appropriate measures of standing and walking, and as such, the SWAT can assist clinicians in setting realistic and timely goals with the individual, monitoring progress, and directing therapeutic interventions and/or priorities.^1^^,^^9^

In 2014, the SWAT was implemented into clinical practice at a subset of Canadian rehabilitation facilities participating in the Rick Hansen SCI Registry (RHSCIR).^13^ RHSCIR is a pan-Canadian prospective, observational registry for SCI, in which collected data include injury, sociodemographics, prehospital/acute/rehabilitation care, as well as community follow-up at 1, 2, and every 5 years thereafter post injury.^13^ At each SWAT-performing site, a trained clinical lead promotes integration of the SWAT into standard care, provides guidance to the site’s physical therapists, and acts as the representative for their site in the Canadian SCI Standing and Walking Measures Group. Since its development, a key question was to evaluate the potential of the SWAT to provide clinicians with insight into an individual’s walking potential after rehabilitation discharge.

We aimed to (1) determine if the SWAT stage and associated measures at rehabilitation discharge could predict community outdoor walking capacity 1-year post-discharge and (2) compare the ability of the SWAT stage and/or measures to predict if those able to walk outdoors would require a mobility aid. Results from this study could facilitate discharge planning, such as equipment prescription and/or the need for ongoing rehabilitation.

## Methods

### Database

This retrospective study used RHSCIR participant data obtained from SWAT-performing rehabilitation facilities between 2014 and 2020. All RHSCIR sites obtained local Research Ethics Board approval before enrolling participants.

### Participants

**Figure 2:**
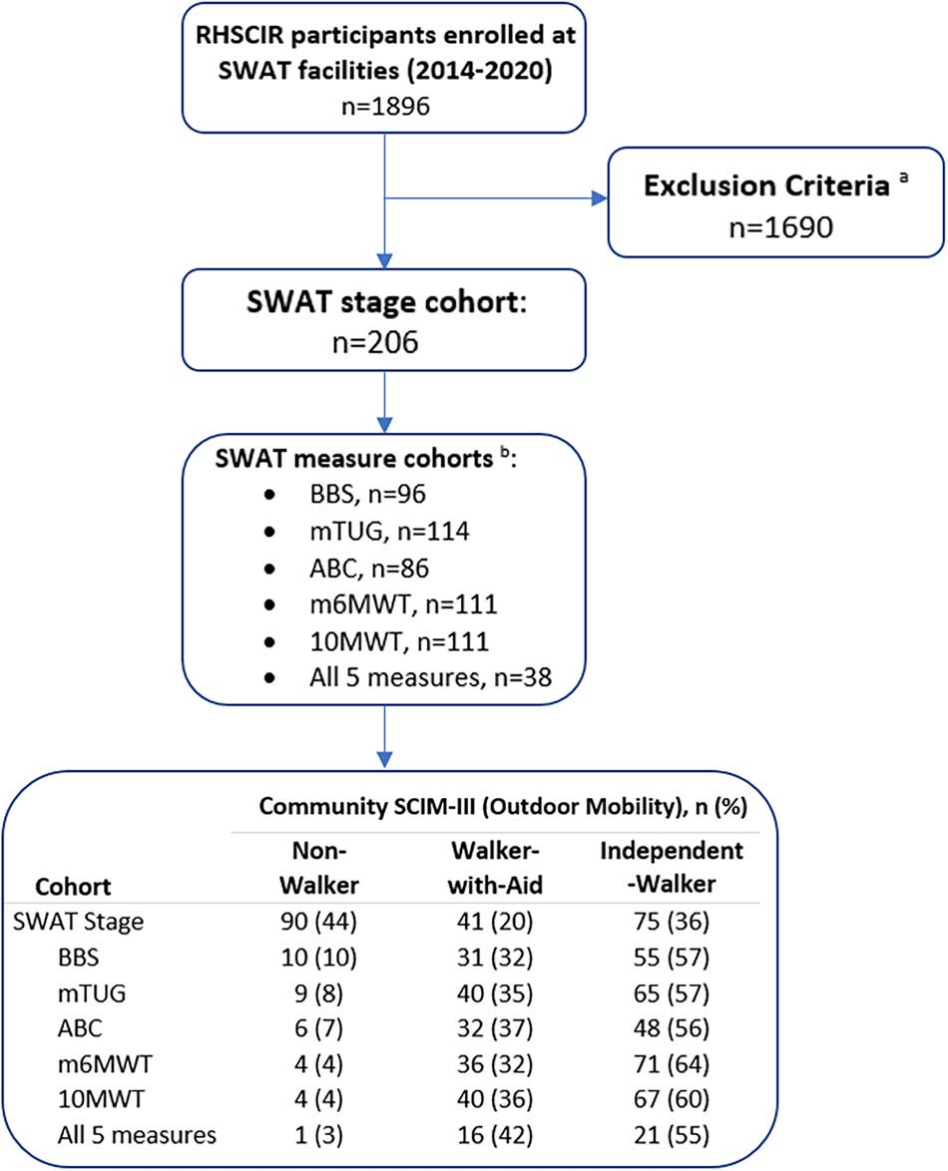
Flow chart of participants in the Standing and Walking Assessment Tool (SWAT) cohort study from the Rick Hansen Spinal Cord Injury Registry (RHSCIR) database from 2014 to 2020. *^a^*Exclusion included: died (*n* = 4); <16 yr old (*n* = 3); SWAT stage missing (*n* = 707); SWAT measure performed >2 wk before discharge (*n* = 199); Not consented to follow-up (*n* = 349); SCIM III data not available (*n* = 326); and community SCIM III data outside 12 (±4) mo after discharge window (*n* = 102). *^b^*SWAT Cohorts derived from the SWAT Stage Cohort. SCIM III = Spinal Cord Independence Measure III; ABC = Activities-Specific Balance Confidence Scale; BBS = Berg Balance Scale; Community nonwalkers = SCIM III outdoor mobility score of 0–3; Community walkers with aids = SCIM III outdoor mobility score of 4–6; Independent community walkers = SCIM III outdoor mobility score of 7–8; m6MWT = modified 6-Minute Walk Test; mTUG = modified Timed “Up & Go”.

From 2014 to 2020, 1896 RHSCIR participants attended 11 SWAT-performing rehabilitation facilities. Of these, 206 participants met the inclusion criteria: ≥16 years old with a TSCI, SWAT staging and measures available within 2 weeks of discharge, and Spinal Cord Independence Measure III (SCIM III) completed 12 (±4) months post rehabilitation discharge. As each SWAT measure was only performed on a subset of participants who met that SWAT stage threshold, the 7 SWAT cohorts (Stage, BBS, mTUG, ABC, m6MWT, 10MWT, and the combination of all 5 measures) had variable participant numbers (Fig. 2).

### Independent Variables

Detailed explanation of the SWAT is described in Verrier et al^1^ In brief, the 12 SWAT stages (Fig. 1) are based on the individual’s functional ability to stand and walk (Suppl. Tab. 1).

The BBS, incorporated at SWAT stage ≥1B, is a 14-item performance-based balance scale designed to assess balance and fall risk.^9^ Each item is rated: 0–4, 0 being unable to perform the task and 4 being able to perform the task independently. A total score from 0 to 56 points reflects balance ability. Additionally, we evaluated BBS subcategories: sitting (item 3), standing (items 2, 6, 7, 8, 9, 10, 13, and 14), and dynamic (items 1, 4, 5, 11, and 12) balance.^14^

The mTUG, incorporated at SWAT stage ≥2A, is a clinical measure for mobility.^10^ Individuals are instructed to rise from a standard armchair, walk 3 meters (m), turn around, and walk back to sit down at their preferred walking speed.^10^ The time required to complete the task and the assistance rating from 1 to 6 (1 being independent, and 6 being unable to complete the task) are recorded, and the task score is calculated in alignment with the SCI Functional Ambulation Profile.^15^

The ABC scale, incorporated at SWAT stage ≥2A upon discharge, is a self-reported measure of balance confidence when performing 16 standing and walking activities incorporating static, dynamic, proactive, and reactive balance.^11^ Individuals are asked to rate their confidence from 0 (no confidence) to 100% (completely confident) in performing the tasks without losing balance or experiencing a sense of unsteadiness.

The m6MWT, incorporated at SWAT stage ≥3B, is used to measure walking endurance by assessing the distance (meters) walked over 6 minutes.^9^^,^^10^ Individuals may use a mobility aid and are allowed rest in standing as needed without stopping the timer and continue walking when able. The 2, 4, and 6-minute distances as well as the Borg Rating of Perceived Exertion (RPE) are recorded. The number of rests or resting time is not recorded. The RPE is a subjective, self-performed rating scale ranging from 6 (no exertion at all) to 20 (maximal exertion),^16^ and is considered a valid and inexpensive tool for monitoring exercise intensity.^17^

The 10MWT, incorporated at SWAT stage ≥3B, is used to assess walking speed; individuals are asked to walk at their preferred speed and then a second time at their maximum speed along a 14-meter walkway with the middle 10 meters being timed,^12^ and both walking speed and reserve are calculated.

### Dependent Variable

To define walking capacity 12 (±4) months post-discharge, the SCIM III item 14 outdoor mobility (> 100 meters) was used.^18^ A score of 0 (total assistance) to 3 (requires supervision while walking with/without devices) defined community nonwalkers, and a score of 4 (uses a walking frame or crutches) to 8 (walks without walking aids) defined community walkers. Furthermore, a score of 4–6 defined community walkers with aids and 7–8 defined independent community walkers (Suppl. Tab. 2). Those using a leg orthosis only were considered independent community walkers.

### Statistical Analyses

Descriptive statistics were calculated for categorical variables with mean and standard deviation (SD), or a median and interquartile ranges (IQR) for continuous variables among the community ambulatory groups: community nonwalkers versus community walkers, and community walkers with aids versus independent community walkers. Bivariate analysis (Student *t*-tests or Wilcoxon Rank Sum Test for continuous variables and Chi-square tests for categorical variables) identified significant differences between variables in the ambulatory groups.

For study aim 1, bivariate analyses were conducted to determine whether statistical associations existed between SWAT stages and measures in persons who were community nonwalkers versus community walkers, and community walkers with aids versus independent community walkers. For study aim 2, multivariable regression analyses were conducted to explore which SWAT measures at discharge were predictors of independent walking in the community. Variables that were statistically significant at the bivariate analyses were included in multivariable analyses ie, age at injury, discharge ASIA Impairment Scale (AIS),^19^ discharge neurological level of injury (NLI),^20^ discharge lower extremity motor score (LEMS), rehabilitation length of stay (LOS), time since discharge, and the SWAT measures.

Using the cohort with all 5 measures (*n* = 38), a receiver operating characteristic (ROC) curve analysis^21^ was completed to evaluate the ability of each SWAT measure to discriminate community walkers with aids from independent community walkers.^22^ All statistical analyses were conducted using SAS software, version 15.1 (SAS Institute Inc, Cary, NC, USA.)

### Role of the Funding Source

The funders played no role in the design, conduct, or reporting of this study.

## Results

Of 206 participants, 96 (47%) had BBS, 114 (55%) had mTUG, 86 (42%) had ABC scale, 111 (54%) had m6MWT, 111 (54%) had 10MWT, and 38 (18%) had all 5 measures (Fig. 2).

### SWAT Stage

The mean (SD) age at injury was 48 (18) years, 157 (76%) were male, 121 (65%) participants were discharged with AIS C/D, the median (IQR) LOS in rehabilitation was 60 (50) days, and the median (IQR) LEMS and SCIM III total mobility score was 42 (48.5) and 25 (18), respectively (Tab. 1). Using the SCIM III outdoor mobility score, 90 (43.7%) participants were classified as community nonwalkers, 116 (56.3%) participants were community walkers; of which, 41 (35.3%) participants were community walkers with aids and 75 (64.7%) participants were independent community walkers.

**Table 1:** Characteristics of the Standing and Walking Assessment Tool (SWAT) Stage Cohort*^a^*

	SCIM III Outdoor Walker Status at CFU, *n* (%)						
Variable	Total *N* = 206	Nonwalkers *N* = 90	Walkers *N* = 116	*P*	Walkers With Aids *N* = 41	Independent Walkers *N* = 75	*P*
Age at injury (y), mean (SD)	48 (18)	47 (29)	52 (30)	.030*^b^*	54 (28)	50 (32)	.767
Sex (male), *n* (%)	157 (76.2)	87 (96.7)	70 (60.3)	.642	28 (68.3)	59 (78.7)	.217
Time post injury (mo), median (IQR)	14 (2)	14 (4)	14 (2)	.010*^b^*	14 (2)	14 (2)	.959
Time post rehab (mo), median (IQR)	10 (2)	10 (2)	11 (2.5)	.032*^b^*	10 (2)	11 (2)	.028*^b^*
Rehab LOS (d), median (IQR)	60 (50)	86 (46)	44 (31)	<.001*^b^*	52 (33)	40 (29)	.003*^b^*
Injury severity at D/C, *n* (%)	186	85	101	<.001*^b^*	35	66	.003*^b^*
AIS A/B	64 (34.4)	55 (64.7)	9 (8.9)		7 (20)	2 (3)	
AIS C/D	121 (65)	30 (35.3)	91 (90.1)		27 (77.1)	64 (97)	
AIS E	1 (0.5)	0	1 (1)		1 (2.9)	0	
NLI at D/C, *n* (%)	193	89	104	.476	38	66	.016*^b^*
C1–T6	125 (64.8)	60 (67.4)	65 (62.5)		18 (47.4)	47 (71.2)	
T7 and below	68 (35.2)	29 (32.6)	39 (37.5)		20 (52.6)	19 (28.8)	
LEMS at D/C, median (IQR)	42 (48.5)	0 (15)	48 (6)	<.001*^b^*	45 (7)	49.5 (3)	.001*^b^*
SCIM III total mobility at D/C, median (IQR)	25 (18)	14 (8)	32 (10)	<.001*^b^*	27 (8)	34 (8)	<.001*^b^*
SWAT stage at D/C, *n* (%)							
0	20 (9.7)	20 (22.2)	0	<.0001*^b^*	0	0	<.0001*^b^*
0.5	34 (16.5)	34 (37.8)	0		0	0	
1A	17 (8.3)	17 (18.9)	0		0	0	
1B	3 (1.5)	2 (2.2)	1 (0.9)		1 (2.4)	0	
1C	1 (0.5)	1 (1.1)	0		0	0	
2A	3 (1.5)	2 (2.2)	1 (0.9)		0	1 (1.3)	
2B	1 (0.5)	1 (1.1)	0		0	0	
2C	3 (1.5)	2 (2.2)	1 (0.9)		0	1 (1.3)	
3A	11 (5.3)	7 (7.8)	4 (3.4)		1 (2.4)	3 (4.0)	
3B	20 (9.7)	3 (3.3)	17 (14.7)		13 (31.7)	4 (0.53)	
3C	59 (28.6)	1 (1.1)	58 (50.0)		25 (61.0)	33 (44.0)	
4	34 (16.5)	0 (0)	34 (29.3)		1 (2.4)	33 (44.0)	
SWAT stage change Adm to D/C,*^c^* median (IQR)	2 (4)	0 (2)	3 (4)	<.001*^b^*	3 (5)	3 (4)	.429
SWAT stage cut-offs, *n* (%)							
0–3A	93 (45.1)	86 (95.6)	7 (6.0)	<.0001*^b^*	2 (4.9)	5 (6.7)	1.000
≥3B	113 (54.9)	4 (4.4)	109 (94.0)		39 (95.1)	70 (93.3)	
0-3B	113 (54.9)	89 (98.9)	24 (20.7)	<.001*^b^*	15 (36.6)	9 (12)	.002*^b^*
≥3C	93 (45.1)	1 (1.1)	92 (79.3)		26 (63.4)	66 (88)	
0–3C	172 (83.5)	90 (100)	82 (70.7)	<.0001*^b^*	40 (97.6)	42 (56)	<.0001*^b^*
4	34 (16.5)	0 (0)	34 (29.3)		1 (2.4)	33 (44)	

^a^ AIS = the American Spinal Injury Association Impairment Scale; CFU = community follow-up 12 (±4) mo after discharge; D/C = rehabilitation discharge; IQR = interquartile range; LEMS = lower extremity motor score; LOS = length of stay; NLI = neurological level of injury; SCIM III = Spinal Cord Independence Measure III.

^b^ Indicates statistical significance

^c^ SWAT stage change from rehabilitation admission to discharge includes the 12 SWAT stages from Stage 0 to 4.

**Figure 3:**
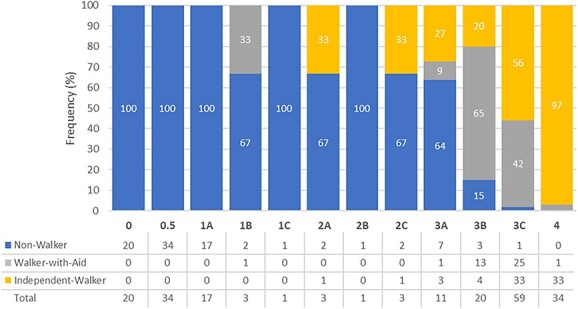
The Standing and Walking Assessment Tool (SWAT) stages at discharge vs the frequencies of participants classified as community nonwalkers, community walkers with aids, and independent community walkers based on the Spinal Cord Independence Measure III outdoor mobility score taken at community follow-up 12 (±4) mo after discharge.

Community nonwalkers were significantly younger, primarily AIS A/B, had lower LEMS, lower SCIM III total mobility scores, and were more likely to be discharged at the lower SWAT stages than community walkers (Tab. 1 and Fig. 3). Correspondingly, community walkers made significant advances in the SWAT stages, their median (IQR) SWAT stage change from admission to discharge was 3 (4) levels, compared to 0 (2) for community nonwalkers. When comparing community walkers with aids and independent community walkers (Tab. 1), community walkers with aids were primarily discharged at SWAT stage 3C (25 out of 41, 78%), whereas independent community walkers were equally discharged at SWAT stage 3C or 4 (33 out of 75, 39%), (*P* < .001). However, there was no significant difference between community walkers with aids and independent community walkers in the SWAT stage change from admission to discharge. Overall, three SWAT stage cutoffs could significantly distinguish community outdoor walking capacity: 0–3A was associated with community nonwalkers; stage 3B or more was associated with community walking (with/without an aid); and stage 4 was associated with independent community walkers (Tab. 1 and Fig. 3).

### SWAT Measures

For SWAT measures at discharge, only community walkers with aids versus independent community walkers were compared. With exception of age at injury and SWAT stage change from admission to discharge, all other demographic and neurological variables significantly differed among community walkers with aids and independent community walkers (Suppl. Tab. 3). Independent community walkers were more likely to have an AIS D injury as well as higher LEMS and SCIM III total scores at discharge. Moreover, their rehabilitation LOS was significantly shorter compared to community walkers with aids. Detailed below, independent community walker participants performed significantly better in all 5 SWAT measures when compared to community walkers with participants with aids (Tab. 2).

**Table 2:** The Standing and Walking Assessment Tool (SWAT) Measures Cohorts*^a^*

**SWAT Measure**	**Variables**	**Total**	**Community Walkers** **With Aids**	**Independent Community Walkers**	***P***
**BBS**	**Sample size, *n***	86	31	55	
	Total score, median (IQR)	53 (10)	46 (29)	55 (5)	<.001*^b^*
	Ceiling effect, *n* (%)	26 (30.2)	3 (9.7)	23 (41.8)	.002*^b^*
	**Sub-categories, median (IQR)**				
	Sitting balance (Item 3)	4 (0)	4 (0)	4 (0)	.203
	Standing balance (Items: 2, 6, 7, 8, 9, 10, 13, and 14)	30 (8)	24.5 (18)	32 (3)	<.001*^b^*
	Dynamic balance (Items: 1, 4, 5, 11, and 12)	19 (4)	16 (8)	20 (2)	<.001*^b^*
**mTUG**	**Sample size, *n***	105	40	65	
	Time (s), median (IQR)	12.7 (9.8)	16.7 (16.7)	10.9 (5.2)	<.001*^b^*
	Assistance rating (median, IQR)	1 (2)	4 (2)	1 (1)	<.001*^b^*
	Task score (median, IQR)	1.8 (5.1)	6.6 (9.3)	1.2 (1.4)	<.001*^b^*
**ABC**	**Sample size, *n***	80	32	48	
	Total score (%), mean (SD)	71.7 (20.1)	62.2 (14.3)	81.9 (11.8)	<.001*^b^*
**m6MWT**	**Sample size, *n***	107	36	71	
	6-Minute distance (m), median (IQR)	334 (201)	260.4 (161.3)	369 (165)	<.001*^b^*
	Borg RPE Scale; median (IQR)	11 (3)	12 (2.5)	11 (3)	.009*^b^*
	Use of walking aid, yes; *n* (%)	60 (56.1)	29 (80.6)	31 (43.7)	<.001*^b^*
**10MWT**	**Sample size, *n***	107	40	67	
	Preferred speed, time (s), mean (SD)	11.6 (7.6)	14.9 (9.0)	9.3 (5.4)	<.001*^b^*
	Preferred speed, gait (m/s), mean (SD)	0.9 (0.6)	0.7 (0.4)	1.1 (0.5)	<.001*^b^*
	Max speed, time (s)	8.6 (4.7)	10.8 (6.4)	7.1 (3.7)	<.001*^b^*
	Max speed, gait (m/s), mean (SD)	1.2 (0.7)	0.9 (0.5)	1.4 (0.7)	<.001*^b^*
	Walking reserve (preferred time—max time)	2.6 (2.2)	3.4 (2.7)	2.4 (1.6)	.014*^b^*
	Use of walking aid, yes; *n* (%)	57 (53.3)	30 (75)	27 (40.3)	<.001*^b^*

^a^ 10MWT = 10-Meter Walk Test; ABC = Activities-specific Balance; BBS = Berg Balance Scale; m6MWT = modified 6-Minute Walk Test; mTUG = modified Timed “Up & Go”; RPE = rating of perceived exertion.

^b^ Indicates statistical significance.

#### B‌BS Cohort

At discharge, independent community walkers had significantly higher BBS total, standing, and dynamic balance scores compared to community walkers with aids (Tab. 2), suggesting that independent community walkers had better balance and lower fall risk. Additionally, the proportion of participants who reached the maximum BBS score was significantly higher in independent community walkers compared to community walkers with aids (42 vs 10%).

#### mTUG Cohort

Independent community walkers performed the mTUG significantly faster at discharge, required little/no assistive device, and had low task scores compared to community walkers with aids, indicating they had better mobility (Tab. 2).

#### ABC Cohort

The median ABC scale score was significantly higher in independent community walkers compared to community walkers with aids, suggesting independent community walkers had high balance confidence at rehabilitation discharge (Tab. 2).

#### m6MWT Cohort

By discharge, independent community walkers could walk 109 meters further than community walkers with aids during the m6MWT (*P* < .001) (Tab. 2). Moreover, the Borg RPE score and use of a walking aid differed significantly between community walkers with aids and independent community walkers. Of note, 31 of 71 (44%) independent community walkers required an aid during the m6MWT at rehabilitation discharge but did not require an aid 1-year later in the community.

#### 10MWT Cohort

At discharge, both the preferred speed and max speed were significantly faster for independent community walkers compared to community walkers with aids (Tab. 2). Additionally, 27 of 67 (40%) independent community walkers required a walking aid during the 10MWT at discharge but did not require an aid 1-year later in the community.

### Predicting Independent Walking Using SWAT Measures

All SWAT measures at discharge, except for the m6MWT, were significantly associated with independent community walkers. Furthermore, subcategories within certain SWAT measures performed better than others, eg, the BBS dynamic balance odds ratio was 1.87 (95% CI = 1.13–3.10), whereas the odds ratio was 1.22 (95% CI = 1.01–1.46) for BBS standing balance.

### Discriminative Ability of the SWAT Measures

The ROC analysis for each SWAT measure had an area under the curve greater than 0.75, indicating fair ability to discriminate community walkers with aids from independent walkers (Suppl. Fig. 1). However, the ABC scale had the highest area under the curve of 0.91 (95% CI = 0.81–1.00), indicating excellent discriminative ability.

## Discussion

The development and implementation of the SWAT ensure that physical therapists can use standardized outcome measures at uniform times to assess the standing and walking functions of patients undergoing inpatient rehabilitation, along with any additional measures based on the physical therapist’s or facility’s requirements. The adoption of SWAT has been facilitated due to the engagement of physical therapists, a focus on feasibility, supported implementation, educational tools, engagement of the Canadian SCI Standing and Walking Measures Group, and the use of RHSCIR to facilitate the collection, reporting, and pooling of SWAT data to answer clinical questions. Furthermore, the SWAT supports a program’s ability to objectively assess the impacts of evolving technologies, equipment, and therapeutic approaches on the rehabilitation of those living with an SCI.^23^

In this study, we identified that SWAT stages 0–3A were significantly associated with community nonwalking, stage 4 was significantly associated with independent community walking (Fig. 3). Furthermore, community nonwalkers were mainly AIS A/B, whereas independent community walkers were mainly AIS C/D at discharge (Tab. 1). Similarly, other studies have found that persons classified as AIS A have limited capacity to regain functional walking, whereas persons classified as AIS C/D have a better prognosis for walking recovery.^24–27^ Thus, the SWAT stages at discharge can be used as an indicator of how well a patient may regain functional walking capacity.

Predictably persons who were discharged at stage 3B were more likely to be community walkers with aids (65%), and persons discharged at stage 3C had the potential to be either community walkers with aids (42%) or independent community walkers (56%) (Fig. 3). It would be interesting to explore this subgroup to further investigate what factors may/may not impact the use of a walking-aid or walking independently in the community. For example, were there ongoing therapies, secondary health conditions such as pain or spasticity, or other environmental or social factors that contributed to their walking ability? Unfortunately, the sample size for this group was too small to explore these factors further.

When reviewing SWAT stage changes, several participants made significant progress going from stage 0 to 1A at admission to reach stage 3B or 3C by discharge (Suppl. Fig. 2A and B). This suggested that they may have the ability to walk with an aid in the community at discharge. They did not, however, maintain this ability to walk with an aid once in the community. For example, of those starting rehabilitation at SWAT stage 0, 2 were discharged at stage 3B and 1 was discharged at stage 3C, but all were community nonwalkers at follow-up (Suppl. Fig. 2B). Interestingly, only 5.3% of the SWAT participants were discharged at SWAT stages 1B–2C, compared to 34.5% at stages 0–1A or 60.2% at stages 3A–4 (Suppl. Fig. 2A). These findings align with the larger SWAT cohort,^8^ as well as the 2020 RHSCIR Annual Report,^28^ which reported that 42% of individuals with TSCI were walking independently with or without an aid by discharge.

It is likely that our method of classifying walking capacity using the SCIM III outdoor mobility score does not represent the person’s physical ability but instead their chosen method of moving outdoors. Alternatively, the systematic review and meta-analysis by Khan et al^29^ reported that 78% of ambulators and 69% of wheelchair users fell at least once per year, or the report by Jørgensen et al^30^ found that almost 50% of community-dwelling ambulatory individuals with TSCI had recurrent falls. The fear of falling could explain why persons with the ability to walk with an aid would chose to use a wheelchair. Secondary health conditions, such as chronic pain and/or spasticity, may also impact functional ability, community participation, as well as quality of life.^31^ In our study, of the stage 3 community nonwalker participants (*n* = 9), many reported having neuropathic pain (100%), spasticity (100%), and fatigue (78%) at community follow-up. Therefore, it is reasonable to suggest that despite having the ability to walk with an aid, using a wheelchair when outdoors may be perceived as being a more efficient and safer form of mobility.

Another consideration may be the physical environment after discharge: urban versus rural settings. Some participants may be able to ambulate with an aid outdoors around the rehabilitation center; however, upon returning home to a more rural location encounter unpaved walkways, steep hills, or buildings that are spaced quite far apart, and may choose to use a wheelchair. In this study, we did not review the discharge destination or its geographical location, and thus future studies should consider this variable.

As each SWAT measure has been demonstrated as reliable, valid, and able to discriminate balance and walking ability in persons with incomplete SCI at a specific time-point,^10^^,^^32–34^ it was encouraging to see that at discharge, these measures could also predict independent walking 1-year post-discharge. Independent community walkers had significantly better balance (BBS and mTUG), were more confident (ABC scale), and could walk further (m6MWT) and faster (10MWT) at discharge than community walker with aids participants (Tab. 2), suggesting that walking ability was maintained following discharge. Furthermore, multivariable regression analyses considering confounding variables such as time since discharge, rehabilitation LOS, AIS, NLI, and LEMS at discharge confirmed that the discharge scores for 4 SWAT measures: BBS, mTUG, ABC scale, and 10MWT significantly correlated with independent community walking (Tab. 3). Additionally, subcategories within measures had a stronger association than others (eg, the BBS dynamic balance score had an odds ratio of 1.9, compared to the BBS standing balance score with an odds ratio of 1.2). These findings align with other studies,^35–37^ validating the use of these measures in identifying balance and walking ability in persons living with SCI.

**Table 3:** Multivariable Regression Analysis to Predict Independent Walking 1 Yr Post-Rehabilitation Discharge for Each Standing and Walking Assessment Tool (SWAT) Measure*^a^*

**SWAT Measure**	**Independent Variables** *^b^*	**Estimate**	**Std Error**	**Odds Ratio**	**95% Wald CI**	***P***
**BBS**	Total score	0.18	0.08	1.20	1.02, 1.40	.025*^c^*
	Standing balance (Items: 2, 6, 7, 8, 9, 10, 13, and 14)	0.20	0.09	1.22	1.01, 1.46	.037*^c^*
	Dynamic balance (Items: 1, 4, 5, 11, and 12)	0.63	0.26	1.87	1.13, 3.10	.014*^c^*
**mTUG**	Task score	−0.48	0.17	0.62	0.44, 0.86	.005*^c^*
**ABC**	Total score	0.13	0.04	1.14	1.05, 1.24	.003*^c^*
**m6MWT**	6-minute distance (m)	0.00	0.00	1.00	1.00, 1.01	.175
	Borg RPE score	−0.13	0.15	0.88	0.65, 1.19	.398
**10MWT**	Preferred speed (m/s)	3.26	1.22	26.2	2.39, 286.3	.008*^c^*
	Max speed (m/s)	2.08	0.87	8.01	1.46, 43.84	.017*^c^*
	Walking reserve	−0.23	0.14	0.80	0.60, 1.05	.109

^a^ 10MWT = 10-Meter Walk Test; ABC = Activities-specific Balance Confidence Scale; AIS = American Spinal Injury Association Impairment Scale; BBS = Berg Balance Scale; LEMS = lower extremity motor score; LOS = length of stay; m6MWT = modified 6-Minute Walk Test; mTUG = modified Timed “Up & Go”; NLI = neurological level of injury; RPE = rating of perceived exertion.

^b^ Each model also included adjustments for the following independent variables: time since discharge (mo), AIS at discharge = A/B vs C/D, NLI at discharge = C1-T7 vs T7 below, LEMS at discharge. Rehab LOS was included in all but the BBS model (see Suppl. Tab. 4).

^c^ Indicates statistical significance

Interestingly, in the bivariate analyses (Tab. 1), both AIS and LEMS were significantly associated with community walkers with or without an aid. However, in the multivariable analysis, after adjusting for time since discharge, AIS, NLI, LEMS, and each SWAT measure at discharge (Tab. 3 and Suppl. Tab. 4), both AIS and LEMS were not found to be significantly associated with independent outdoor walking in the community. Similarly, in the study by van Middendorp et al,^38^ they found that AIS conversion poorly correlated with the ability to walk in persons with TSCI when compared with walking indices such as the TUG and 10MWT.

Notably in our study, 26 (30%) participants reached the max BBS score. Similarly, the BBS ceiling effect has been reported in other SCI studies,^9^^,^^39^^,^^40^ suggesting that the BBS test lacked the sensitivity to discriminate individuals with minimal balance limitations.^41^ Nevertheless, in the multivariable logistic regression analyses, both the BBS total score and the subcategories were associated with independent community walking (Tab. 3); and in the cohort with all 5 measures, the BBS (total score) could distinguish community walkers with aids from independent community walkers with an area under the curve of 0.77 (Suppl. Fig. 1).

Balance ability as measured by BBS and mTUG was also evident in the self-reported ABC scale. A high ABC scale was significantly associated with independent community walking (Tab. 3), and in the cohort with all 5 measures, the area under the curve of 0.91 indicated that the ABC scale could best distinguish community walkers with aids from independent community walkers (Suppl. Fig. 1). Reasonably, a person who has greater confidence and balance control after SCI will be better able to navigate the complexities of walking outdoors. Similarly, Shah et al^11^ found that self-reported balance confidence was associated with higher levels of postural/balance control. The ABC scale score was also found to correlate with the 10MWT walking speed.^11^ In this study, independent community walker participants walked 1.5 times faster (10MWT) and 109 meters further (m6MWT) than community walkers with aids; additionally, the mean preferred walking speed at discharge was 1.1 m/s for independent walkers and 0.7 m/s for walkers with aid. These findings are in agreement with van Silfhout et al,^42^ which suggested a walking speed of 0.59 m/s as the cutoff between persons who do and do not ambulate independently in the community.

At discharge, several participants used a walking aid when performing the m6MWT (*n* = 60) and 10MWT (*n* = 57), and approximately 40% of these participants became independent community walkers upon follow-up (Tab. 2). On further exploration of the type of aid being used at rehabilitation discharge, there was no clear indication which was more predominant. Some studies have reported that long-lasting use of walking aid may introduce negative consequences such as abnormal walking patterns^43^ and/or upper limb pain/dysfunction, such as shoulder pain due to overuse injury.^44^ This needs to be explored further to identify factors post-discharge that impact individuals to forgo the walking aid and become independent walkers in the community.

Overall, this study highlighted the potential of the SWAT to be used in the clinical setting as a prognosticating method to predict independent outdoor walking ability in the community. This toolkit allows clinicians to test the client’s readiness for walking, as well as to assist in identifying areas of deficit that need to be addressed clinically. Moreover, as SWAT provides a practical framework for decision making regarding which outcome measures to perform and when to preform them, this will ensure standardized use of measures, and the timing there of, which will enhance cross comparison among rehabilitation facilities. The SWAT may also assist with program-level planning concerning inpatient LOSs and outpatient services, particularly regarding the prescription of walking aids.

The main limitation was the relatively small cohort size and the completeness of the SWAT dataset. Due to the novel nature of the SWAT and its staggered implementation, many participants had the SWAT stage data missing/incomplete (*n* = 707). Second, although most motor recovery occurs within 6–9 months and typically plateaus 12–18 months post SCI,^45^ our study used the SCIM III 12 (±4) months post-discharge to define community walking ability due to data collection availability. This means that motor recovery may not have plateaued in all participants. Future work should address these limitations and further investigate the subgroup of participants discharged at stage 3C who gained the ability to walk independently in the community, and the subgroup of participants discharged at/above stage 3B but at follow-up were community nonwalkers. To do this, both current and future rehabilitation facilities should be encouraged to implement the SWAT into their rehabilitation programs, ensure datasets are completed, and explore ways to enhance community follow-up.

## Conclusion

We identified that SWAT stages (3B/higher) and measures (BBS, ABC scale, mTUG, and 10MWT) were associated with the ability to walk (with/without an aid) outdoors independently for long distances 1-year post-discharge. SWAT, a set of outcome measures that predict community walking capacity, can help enable clinicians to enhance discharge planning, determine long-term equipment needs, and establish outpatient follow-up.

## Supplementary Material

2022-0630_r1_swat_supplementary_9mar_final_tsr_pzad106

## Data Availability

The RHSCIR data supporting the findings of this study are available from the corresponding author on request.
