# Supplementary material for: Using the Standing and Walking Assessment Tool at Discharge Predicts Community Outdoor Walking Capacity in Persons With Traumatic Spinal Cord Injury
Source: Phys Ther. 2023 Aug 10;103(11):pzad106. doi: 10.1093/ptj/pzad106 (PMC10799252; doi:10.1093/ptj/pzad106)
Supplement: 2022-0630_r1_swat_supplementary_9mar_final_tsr_pzad106 [file 2022-0630_r1_swat_supplementary_9mar_final_tsr_pzad106.pdf]

Supplementary Figure Legends

Supplementary Figure 1. Receiver-operating characteristic (ROC) curves for predicting independent walking using the Standing and Walking Assessment Tool (SWAT) measures Berg Balance Scale (BBS), modified Timed Up and Go (mTUG), Activities-specific Balance Confidence (ABC) Scale, modified 6-minute Walk Test (m6MWT); 10-meter Walk Test (10MWT). The insert reports the different areas under the curve (AUC) and the corresponding confidence interval (CI).

Supplementary Figure 2. The Standing and Walking Assessment Tool (SWAT) stage at admission and at discharge (**A**) and walking ability 1-year post-discharge using SCIM-III, item 14, outdoor mobility (**B**). To interpret the top-left graph, there were 67 participants who entered rehabilitation at SWAT Stage 0 and by rehabilitation discharge 20 participants remained at stage 0, 26 participants improved to stage 0.5, 8 participants improved to stage 1A, and so forth. The legend indicates the SCIM-III outdoor walking ability in the community: Non-Walkers (blue), Walkers-with-Aid (yellow), or Independent-Walkers (orange).

Supplementary Figure 1

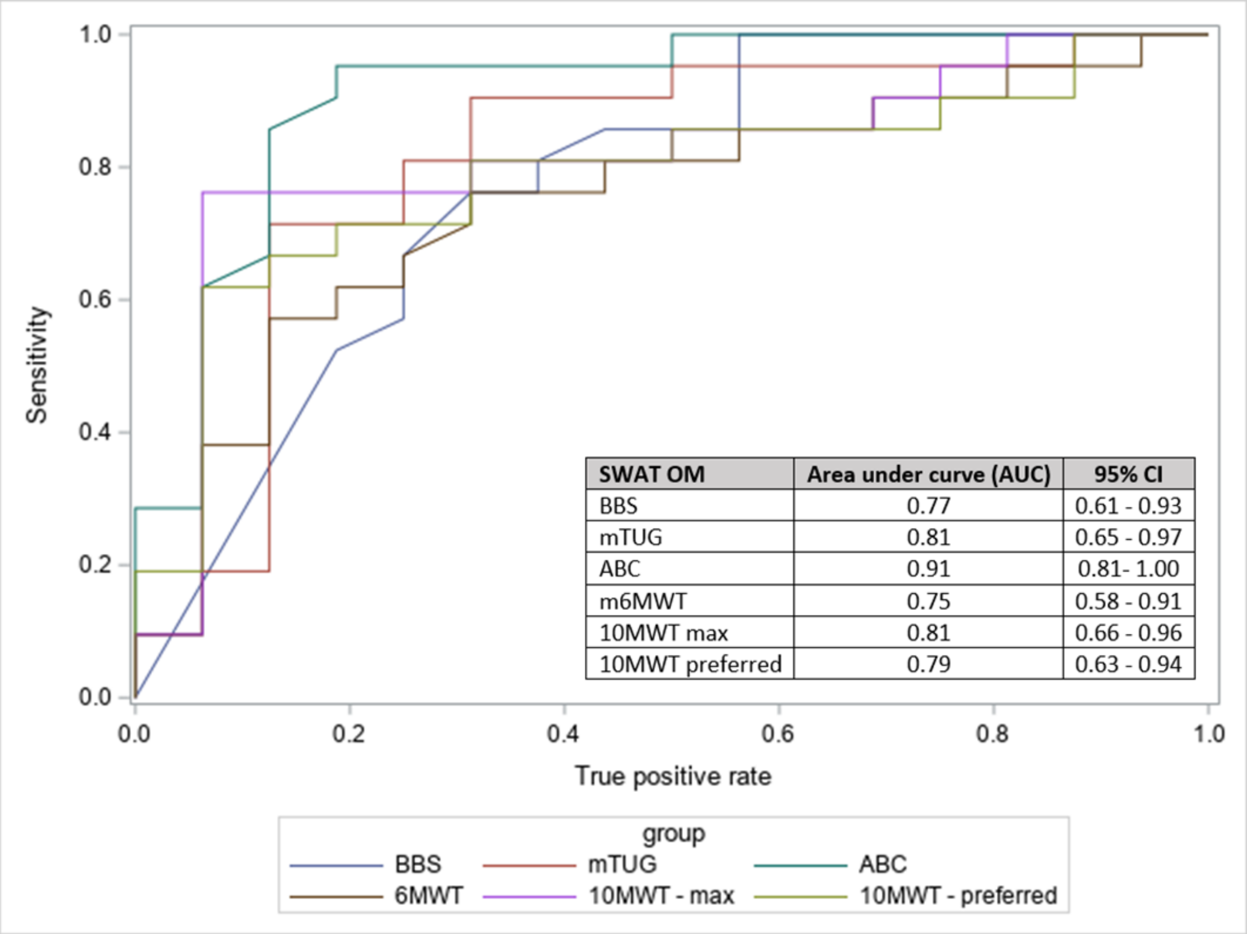

SWAT OM	Area under curve (AUC)	95% CI
BBS	0.77	0.61 - 0.93
mTUG	0.81	0.65 - 0.97
ABC	0.91	0.81- 1.00
m6MWT	0.75	0.58 - 0.91
10MWT max	0.81	0.66 - 0.96
10MWT preferred	0.79	0.63 - 0.94

Supplementary Figure 2A

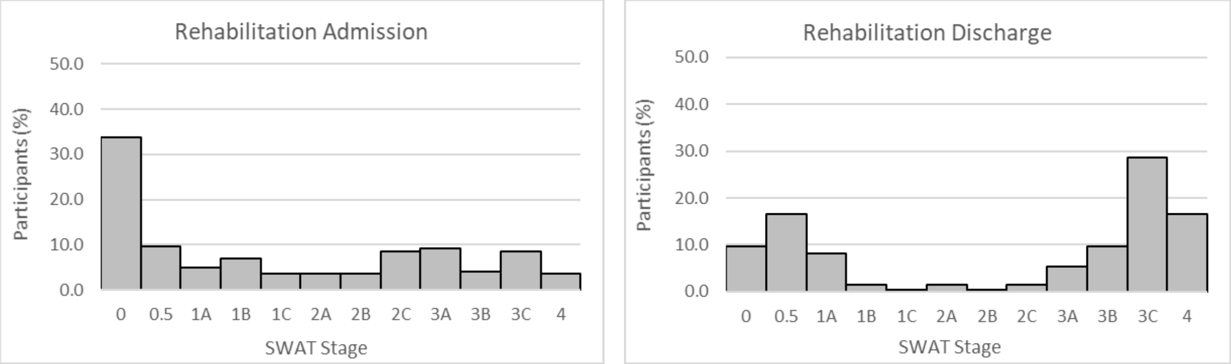

Supplementary Figure 2B

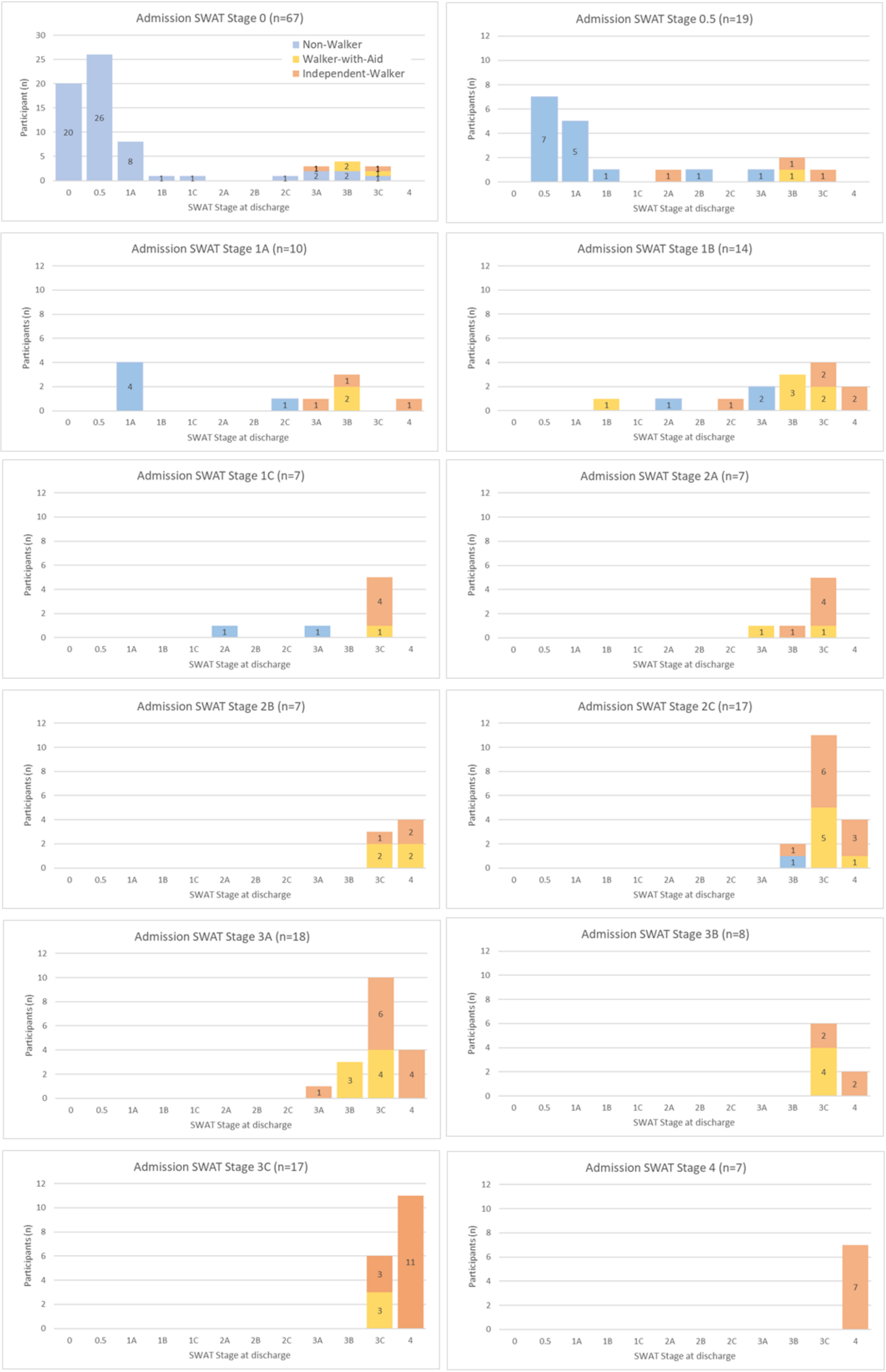

Supplementary Table 1. Standing and Walking Assessment Tool (SWAT) stage definitions (1).

Stage	Description	Definition
**0.0**	No Independent Sitting Capacity	The patient is unable to sit independently on a firm surface with no back support with hips and knees at 90 degrees and feet on the floor for 60 seconds without using arms to stabilize.
**0.5**	Independent Sitting Capacity	The patient is able to sit independently on a firm surface with no back support with hips and knees at 90 degrees and feet on the floor for 60 seconds without using arms to stabilize.
**1**	Standing Capacity	The patient cannot ambulate but may be able to stand with total assistance.
**1A**	Trace LE Movement	Unable to stand without total assistance of gait aid and/or orthoses and/or therapist(s). No voluntary LE functional movement (LE MMTs Gr 1 or less in of the anti-gravity muscles*). In order to be staged as a 1A, the patient must have attempted a stand.<br><br>Examples of total assistance in standing:<br>1. Patient stands at parallel bars with full weight bearing through UEs and minimal weight through the feet.<br>2. Patient stands in parallel bars/walker/forearm crutches with bilateral KAFOs<br>3. Patient stands with a therapist(s) providing total assistance at hips and knees
**1B**	Voluntary Non-Functional LE Movement	Unable to stand independently/needs partial assistance of gait aid and/or orthoses (except bilateral KAFOs) and/or therapist(s) to stand. The use of bilateral KAFOs is not allowed. Voluntary LE Movement (LE MMTs of Gr 3 or higher in less than 4 of the anti-gravity muscles*).<br><br>Example of standing with assistance:<br>1. Patient stands in parallel bars/walker/bedside without total assistance of another person or walking aid and therapist assistance to maintain an upright posture. Patient may have only partial weight bearing through the UEs with weight bearing through the feet. The patient may have LE orthoses on except for Bilateral KAFOs.
**1C**	Voluntary Functional LE Movement	Able to stand independently with minimal assistance of gait aid for a limited amount of time (less than 30 secs). Orthoses are allowed except for bilateral KAFOs. Voluntary LE Movement. (LE MMTs of Gr 3 or higher in 4 or more of the anti-gravity muscles*. Gr 1 Tib. Ant with an orthosis would fit this group.)<br><br>Example of independent standing:<br>1. Patient stands in parallel bars/walker/bedside with occasional UE contact. The patient may have LE orthoses on except for bilateral KAFOs.
**2**	Therapeutic Walking Capacity (Indoors)	The patient is starting to ambulate with therapist assistance and gait aids/orthoses and progresses toward minimal assistance.
**2A**	Max Assist	Ability to stand and initiate reciprocal steps through voluntary LE movement but requires maximal physical assistance (>50% of total effort) of at least one person and may include use of assistive devices/parallel bars/suspension harness and/or orthoses with the exception of bilateral KAFOs.<br><br>Examples of maximal physical assistance:<br>1. Physical assistance is provided to prevent a fall during most attempts at walking.<br>2. Physical assistance of 2 people is required to complete the gait cycle.<br>3. Physical assistance is required for 1 leg, PLUS continuous physical assistance at trunk/safety belt to steady.
**2B**	Mod Assist	Ability to stand and initiate reciprocal steps through voluntary LE movement but requires moderate physical assistance (25-50% of total effort) of one person and may include the use of assistive walking aids and/or orthoses with the exception of the bilateral KAFOs (cannot use parallel bars or suspension harness).<br><br>Examples of moderate physical assistance:<br>1. Physical assistance is required for 1 leg only<br>2. Continuous physical assistance is required at the trunk/safety belt to steady<br>3. Intermittent physical assistance at trunk/safety belt to steady, PLUS assistance with placement of assistive device (e.g., guiding walker).

**2C**	Min Assist	Ability to stand and initiate reciprocal steps through voluntary LE movement but requires minimal physical assistance (<25% of total effort) of one person and may include the use of assistive devices and/or orthoses with the exception of the bilateral KAFOs. (Cannot use parallel bars or suspension harness).<br>Examples of minimal physical assistance:<br>1. Assistance with the placement of assistive device (e.g., guiding walker).<br>2. Intermittent physical assistance is required at trunk/safety belt to steady.
**3**	Functional Walking Capacity (Outdoors)	The patient is starting to ambulate without therapist assistance but still requires gait aids/orthoses. The patient progresses to ambulating in the community.
**3A**	Supervised Household Ambulator	Ability to ambulate daily using reciprocal steps over ground for short distances (10-100m) with supervision. The person may use assistive devices and /or orthoses with the exception of bilateral KAFOs.<br>Examples of supervision:<br>1. Verbal cueing<br>2. Standing close-by in case of a loss of balance, but no physical contact
**3B**	Independent Household Ambulator	Ability to ambulate daily using reciprocal steps over ground for short distances (10-100m) independently. The person may use assistive devices and/or orthoses with the exception of the bilateral KAFOs.
**3C**	Community Ambulator	Ability to ambulate daily using reciprocal steps over ground for long distances (>100m) independently. The person may use assistive devices and/or orthoses with the exception of the bilateral KAFOs.
**4**	Full Walking Capacity	The patient ambulates independently without therapist assistance or gait aids/orthoses. An independent ambulator has the ability to ambulate full time daily at home and in the community without assistive devices, orthoses, or physical assistance.

*Anti-gravity muscles: Tibialis-anterior muscles, Soleus, Quadriceps femoris, Glutei.

'Ability to stand' refers to a patient's ability to maintain static standing, and not go from sitting to standing.

The ability to go from sit to stand is evaluated in a number of measures.

LE, lower extremity; MMTs, manual muscle testing grading system; KAFO, Knee-Ankle-Foot Orthoses; UE, upper extremity (1).

Supplementary Table 2. Walking ability based on the Spinal Cord Independence Measure (SCIM-III), Item 14, Outdoor Mobility (>100meters).

<table>
<tr><th>SCIM-III Outdoor Mobility Score</th><th colspan="2">Community Walking Ability</th></tr>
<tr><td>0. Requires total assistance
1. Needs an electric wheelchair or partial assistance to operate a manual wheelchair
2. Moves independently in a manual wheelchair
3. Requires supervision while walking (with/without devices)</td><td colspan="2">Non-Walkers</td></tr>
<tr><td>4. Walks with a walking frame or crutches (swing)
5. Walks with crutches or two canes (reciprocal waking)
6. Walks with one cane</td><td rowspan="2">Walkers</td><td>Walkers-with-Aid</td></tr>
<tr><td>7. Needs leg orthosis only
8. Walks without walking aids</td><td>Independent-Walkers</td></tr>
</table>

Supplementary Table 3. The Standing and Walking Assessment Tool (SWAT) measures, a comparison of Community-Walkers-with-Aid vs. Community-Independent Walkers.

Variable	BBS				mTUG				ABC				m6MWT				10MWT			
	Total N=86	CWA N=31	CIW N=55	P	Total N=105	CWA N=40	CIW N=65	P	Total N=80	CWA N=32	CIW N=48	P	Total N=107	CWA N=36	CIW N=71	P	Total N=107	CWA N=40	CIW N=67	P
Age at injury (years), median (IQR)	54 (29)	54 (25)	54 (33)	0.765	52 (26)	53 (26)	52 (28)	0.763	49 (29.5)	51.5 (25)	48 (32.5)	0.689	51 (29)	57.5 (29.5)	50 (28)	0.289	50 (28)	51.5 (27.5)	49 (30)	0.794
Time post injury (months), median (IQR)	14 (2)	14 (1)	14 (2)	0.539	14 (2)	14 (1)	14 (2)	0.460	14 (2)	14 (1)	14 (2)	0.539	14 (2)	13.5 (1.5)	14 (2)	0.329	14 (2)	14 (1)	14 (2)	0.266
Time post rehab (months), median (IQR)	10 (2)	10 (2)	11 (3)	**0.012**	10 (2)	10 (2)	11 (2)	**0.008**	10.5 (2)	10 (2)	11 (2.5)	0.051	10 (2)	9.5 (2)	11 (3)	**0.012**	10 (2)	10 (2)	11 (3)	**0.01**
Rehab LOS (days), median (IQR)	45 (37)	50 (43)	43 (29)	0.07	49 (36)	57 (38)	45 (25)	**0.016**	45 (32)	57 (38)	41 (27)	**0.019**	49 (35)	65 (45.5)	45 (26)	**0.002**	49 (35)	57 (37)	45 (28)	**0.026**
Injury severity at D/C, n (%)																				
AIS A/B	4 (5)	3 (12)	1 (2)		6	5 (14)	1 (2)		6	5 (19)	1 (2)		5	4 (13)	1 (2)		8	6 (16.7)	2 (3)	
AIS C	2 (3)	2 (8)	0	**0.007**	3	2 (6)	1 (2)	**0.012**	2	1 (4)	1 (2)	**0.024**	3	2 (6)	1 (2)	**0.024**	3	2 (5.6)	1 (2)	**0.014**
AIS D	67 (91)	19 (76)	48 (98)		80	27 (77)	53 (96)		59	20 (74)	39 (95)		86	26 (81)	60 (97)		83	27 (75)	56 (95)	
AIS E	1 (1.4)	1 (4)	0		1	1 (2.9)	0		1	1 (4)	0		0	0	0		1	1 (2.8)	0	
NLI at D/C, n (%)																				
C1 - T6	48 (63)	12 (43)	36 (75)	**0.005**	62	20 (53)	42 (75)	**0.025**	43	13 (43)	30 (71)	**0.017**	64	18 (55)	46 (72)	0.088	52	10 (53)	42 (69)	0.105
T7 & below	28 (37)	16 (57)	12 (25)		32	18 (47)	14 (25)		29	17 (57)	12 (29)		33	15 (46)	18 (28)		37	18 (47)	19 (31)	
LEMS at D/C, median (IQR)	47 (8)	42 (12)	50 (4)	**<0.001**	48 (7)	44 (10)	49 (5)	**0.006**	48 (8)	43 (11)	49 (4)	**0.005**	48 (7)	42 (12)	49 (5)	**0.002**	47 (7)	46 (14)	49 (6)	**0.027**
SCIM-III at D/C, median (IQR)																				
Total mobility	31 (8)	27 (5)	33 (8)	**<0.001**	31 (7)	27 (7)	33 (6)	**<0.001**	31 (9)	27 (7)	34 (8)	**<0.001**	32 (8)	27 (7)	34 (8)	**<0.001**	31 (8)	27 (7)	34 (8)	**<0.001**
Indoor	6 (3)	5 (1.5)	8 (2)	**<0.001**	6 (3)	5 (2)	8 (2)	**<0.001**	6 (3)	5 (2)	8 (2)	**<0.001**	6 (3)	5 (2)	8 (2)	**<0.001**	6 (3)	5 (2)	8 (2)	**<0.001**
Moderate distance	5 (1)	5 (1)	6 (3)	**<0.001**	6 (2)	5 (2)	6 (3)	**<0.001**	5 (3)	5 (1)	6 (3)	**<0.001**	6 (3)	5 (2)	6 (3)	**<0.001**	6 (4)	5 (2)	6 (3)	**<0.001**
Outdoor	5 (2)	4 (3)	6 (3)	**<0.001**	5 (2)	4 (3)	6 (3)	**<0.001**	5 (2)	4 (3)	6 (3)	**<0.001**	5 (2)	4 (3)	6 (3)	**<0.001**	5 (2)	4 (3)	6 (3)	**<0.001**
SWAT stage at D/C[a], median (IQR)	3 (0)	3 (0)	3 (1)	**0.002**	3 (0)	3 (0)	3 (1)	**<0.001**	3 (0)	3 (0)	3 (1)	**<0.001**	3 (1)	3 (0)	3 (1)	**<0.001**	3 (1)	3 (0)	3 (1)	**<0.001**
SWAT Stage change [a]	1 (1)	1 (2)	1 (1)	0.446	1 (2)	1 (2)	1 (1)	0.37	1 (2)	1 (2)	1 (2)	0.818	1 (1)	1 (2)	1 (1)	0.931	1 (2)	1 (2)	1 (1)	0.301

[a] SWAT stage change from rehabilitation admission to discharge includes the 12 SWAT stages from Stage 0 to 4.

IQR, interquartile range (IQR); LOS, length of stay; AIS, the American Spinal Injury Association Impairment Scale; NLI, neurological level of injury; LEMS, lower extremity motor score; D/C, rehabilitation discharge; SCIM-III, Spinal Cord Independence Measure; CWA, Community-Walkers-with-Aid; CIW, Community-Independent Walkers.

Supplementary Table 4. Multivariable logistic regression analysis to predict Community-Independent-Walking vs. Community-Walking-with-Aid 1-year post-rehabilitation discharge for each Standing and Walking Assessment Tool (SWAT) measure BBS (total, standing balance, dynamic balance), mTUG, ABC (total, indoor, outdoor), m6MWT (distance, Borg RPE), and 10MWT (preferred speed, max speed, walking reserve).

Independent variable	Estimate	Std error	Odds ratio	95% Wald CI	p-value
Time since discharge (months)	0.25	0.27	1.28	0.76, 2.15	0.356
AIS at discharge					
AIS A/B (baseline)					
AIS C/D	0.45	0.83	2.44	0.10, 62.03	0.589
NLI at discharge					
C1 - T6 (baseline)					
T7 & below	-0.22	0.52	0.64	0.08, 4.82	0.663
LEMS at D/C	-0.02	0.10	0.98	0.81, 1.19	0.845
**BBS, Total Score**	0.18	0.08	1.20	1.02, 1.40	**0.025**

Independent variable	Estimate	Std error	Odds ratio	95% Wald CI	p-value
Time since discharge (months)	0.28	0.26	1.33	0.79, 2.21	0.281
AIS at discharge					
AIS A/B (baseline)					
AIS C/D	0.57	0.81	3.13	0.13, 74.16	0.479
NLI at discharge					
C1 - T6 (baseline)					
T7 & below	-0.14	0.54	0.76	0.09, 6.43	0.802
LEMS at D/C	0.01	0.09	1.01	0.85, 1.21	0.877
**BBS, Standing Category**	0.20	0.09	1.22	1.01, 1.46	**0.037**

Independent variable	Estimate	Std error	Odds ratio	95% Wald CI	p-value
Time since discharge (months)	0.22	0.27	1.25	0.74, 2.11	0.411
AIS at D/C					
AIS A/B (baseline)					
AIS C/D	0.32	0.96	1.89	0.04, 80.25	0.741
NLI at D/C					
C1 - T6 (baseline)					
T7 & below	-0.02	0.57	0.97	0.10, 9.22	0.978
LEMS at D/C	-0.04	0.11	0.96	0.78, 1.19	0.730
**BBS, Dynamic Category**	0.63	0.26	1.87	1.13, 3.10	**0.014**

Independent variable	Estimate	Std error	Odds ratio	95% Wald CI	p-value
Time since D/C (months)	0.09	0.19	1.10	0.75, 1.6	0.629
AIS at D/C					
AIS A/B (baseline)					
AIS C/D	1.11	0.81	9.16	0.38, 222.02	0.174
NLI at D/C					
C1 - T6 (baseline)					
T7 & below	0.20	0.48	1.49	0.23, 9.79	0.680
LEMS at discharge	-0.08	0.09	0.92	0.78, 1.09	0.339
Rehab LOS (days)	-0.01	0.02	0.99	0.96, 1.03	0.712

**mTUG, Task Score**	-0.48	0.17	0.62	0.44, 0.86	**0.005**

Independent variable	Estimate	Std error	Odds ratio	95% Wald CI	p-value
AIS at D/C					
AIS A/B (baseline)					
AIS C/D	1.35	0.98	14.90	0.32, 703.28	0.170
NLI at D/C					
C1 - T6 (baseline)					
T7 & below	0.13	0.59	1.29	0.13, 12.77	0.829
LEMS at D/C	0.02	0.09	1.02	0.86, 1.20	0.839
Rehab LOS (days)	0.00	0.02	1.00	0.96, 1.03	0.818
**ABC, Total Score**	0.13	0.04	1.14	1.05, 1.24	**0.003**

Independent variable	Estimate	Std error	Odds ratio	95% Wald CI	p-value
AIS at D/C					
AIS A/B (baseline)					
AIS C/D	1.09	0.90	8.90	0.26, 300.21	0.224
NLI at D/C					
C1 - T6 (baseline)					
T7 & below	-0.06	0.54	0.89	0.11, 7.32	0.910
LEMS at D/C	0.11	0.08	1.11	0.95, 1.31	0.191
Rehab LOS (days)	0.00	0.02	1.00	0.96, 1.03	0.832
**ABC, Indoor Score (items 1 to 2)**	0.13	0.04	1.14	1.04, 1.24	**0.003**

Independent variable	Estimate	Std error	Odds ratio	95% Wald CI	p-value
AIS at D/C					
AIS A/B (baseline)					
AIS C/D	1.05	0.95	8.25	0.20, 346.76	0.269
NLI at D/C					
C1 - T6(baseline)					
T7 & below	-0.27	0.61	0.59	0.05, 6.32	0.659
LEMS at D/C	-0.02	0.09	0.98	0.82, 1.16	0.795
Rehab LOS (days)	0.00	0.02	1.00	0.97, 1.04	0.885
**ABC, Task Score (items 3 to 7)**	0.09	0.03	1.09	1.03, 1.17	**0.006**

Independent variable	Estimate	Std error	Odds ratio	95% Wald CI	p-value
AIS at D/C					
AIS A/B (baseline)					
AIS C/D	1.15	0.99	9.98	0.20, 489.48	0.247
NLI at D/C					
C1 - T6 (baseline)					
T7 & below	-0.43	0.62	0.42	0.04, 4.84	0.489
LEMS at D/C	0.04	0.09	1.04	0.87, 1.23	0.679
Rehab LOS (days)	0.00	0.02	1.00	0.96, 1.04	0.972
**ABC, Outside Score (items 8 to 16)**	0.12	0.04	1.13	1.04, 1.24	**0.006**

Independent variable	Estimate	Std error	Odds ratio	95% Wald CI	p-value
Time since D/C (months)	0.03	0.19	1.03	0.71, 1.51	0.874
AIS at D/C					
AIS A/B (baseline)					
AIS C/D	0.89	0.75	5.87	0.31, 110.30	0.237
LEMS at D/C	0.07	0.06	1.07	0.96, 1.19	0.230
Rehab LOS (days)	-0.01	0.01	1.00	0.97, 1.02	0.699
**m6MWT, 6min distance (m)**	0.00	0.00	1.00	1.00, 1.01	0.175

Independent variable	Estimate	Std error	Odds ratio	95% Wald CI	p-value
Time since D/C (months)	0.14	0.21	1.15	0.76, 1.74	0.514
AIS at D/C					
AIS A/B (baseline)					
AIS C/D	1.36	1.04	15.25	0.26, 882.25	0.188
LEMS at D/C	0.05	0.06	1.05	0.93, 1.18	0.453
Rehab LOS (days)	-0.01	0.01	0.99	0.96, 1.02	0.498
**m6MWT, Borg RPE Score**	-0.13	0.15	0.88	0.65, 1.19	0.398

Independent variable	Estimate	Std error	Odds ratio	95% Wald CI	p-value
Time since D/C (months)	0.02	0.18	1.02	0.72, 1.46	0.903
AIS at D/C					
AIS A/B (baseline)					
AIS C/D	0.67	0.56	3.79	0.42, 34.14	0.234
LEMS at D/C	0.01	0.05	1.01	0.91, 1.12	0.898
Rehab LOS (days)	0.00	0.01	1.00	0.98, 1.03	0.785
**10MWT, Preferred Speed (m/s)**	3.26	1.22	26.17	2.39, 286.33	**0.008**

Independent variable	Estimate	Std error	Odds ratio	95% Wald CI	p-value
Time since D/C (months)	0.04	0.18	1.04	0.73, 1.48	0.842
AIS at D/C					
AIS A/B (baseline)					
AIS C/D	0.67	0.54	3.84	0.46, 31.87	0.213
LEMS at D/C	0.00	0.05	1.00	0.90, 1.11	0.986
Rehab LOS (days)	0.00	0.01	1.00	0.97, 1.02	0.893
**10MWT, Max Speed (m/s)**	2.08	0.87	8.01	1.46, 43.84	**0.017**

Independent variable	Estimate	Std error	Odds ratio	95% Wald CI	p-value
Time since D/C (months)	0.09	0.18	1.10	0.78, 1.54	0.605
AIS at D/C					
AIS A/B (baseline)					
AIS C/D	0.70	0.52	4.09	0.53, 31.77	0.178
LEMS at D/C	0.05	0.05	1.05	0.95, 1.16	0.343
Rehab LOS (days)	0.00	0.01	1.00	0.98, 1.03	0.970
**10MWT, Walking Reserve**	-0.23	0.14	0.80	0.60, 1.05	0.109

BBS, Berg Balance Scale; mTUG, modified Timed Up and Go; ABC, Activities-specific Balance Confidence Scale; m6MWT, modified 6-minute Walk Test; RPE, Rating of perceived exertion; 10MWT, 10-meter Walk Test; D/C, discharge; m/s, meters/second; CI, confidence interval; AIS, the American Spinal Injury Association Impairment Scale; NLI, neurological level of injury; LEMS, lower extremity motor score; LOS, length of stay.
